# A Cloud-Based Virtual Reality App for a Novel Telemindfulness Service: Rationale, Design and Feasibility Evaluation

**DOI:** 10.2196/resprot.6849

**Published:** 2017-06-05

**Authors:** Imre Cikajlo, Ursa Cizman Staba, Suzana Vrhovac, Frances Larkin, Mark Roddy

**Affiliations:** ^1^ University Rehabilitation Institute, Republic of Slovenia Ljubljana Slovenia; ^2^ Mind Myths Ltd Office 9: Sligo Enterprise & Technology Centre Strandhill, Co Sligo Ireland

**Keywords:** Virtual reality, headset, Samsung, psychology, mindfulness, telepsychology, telehealth technology, telemedicine

## Abstract

**Background:**

Worldwide, there has been a marked increase in stress and anxiety, also among patients with traumatic brain injury (TBI). Access to psychology services is limited, with some estimates suggesting that over 50% of sufferers are not accessing the existing services available to them for reasons such as inconvenience, embarrassment, or stigmatization concerns around mental health. Health service providers have increasingly been turning to drug-free therapies, such as mindfulness programs, as complementary treatments.

**Objective:**

Virtual reality (VR) as a new delivery method for meditation-based stress and anxiety reduction therapy offers configurable environments and privacy protection. Our objective was to design a serious learning-meditation environment and to test the feasibility of the developed telemindfulness approach based on cloud technologies.

**Methods:**

We developed a cloud-based system, which consisted of a Web interface for the mindfulness instructor and remote clients, who had 3D VR headsets. The mindfulness instructor could communicate over the Web interface with the participants using the headset. Additionally, the Web app enabled group sessions in virtual rooms, 360-degree videos, and real interactions or standalone meditation. The mindfulness program was designed as an 8-week Mindfulness-Based Stress Reduction course specifically for the developed virtual environments. The program was tested with four employees and four patients with TBI. The effects were measured with psychometric tests, the Mindful Attention Awareness Scale (MAAS) and the Satisfaction With Life Scale (SWLS). Patients also carried out the Mini-Mental State Examination (MMSE). An additional objective evaluation has also been carried out by tracking head motion. Additionally, the power spectrum analyses of similar tasks between sessions were tested.

**Results:**

The patients achieved a higher level of life satisfaction during the study (SWLS: mean 23.0, SD 1.8 vs mean 18.3, SD 3.9) and a slight increase of the MAAS score (mean 3.4, SD 0.6 vs mean 3.3, SD 0.4). Particular insight into the MAAS items revealed that one patient had a lower MAAS score (mean 2.3). Employees showed high MAAS scores (mean 4.3, SD 0.7) and although their SWLS dropped to mean 26, their SWLS was still high (mean 27.3, SD 2.8). The power spectrum showed that the employees had a considerable reduction in high-frequency movements less than 0.34 Hz, particularly with the 360-degree video. As expected, the patients demonstrated a gradual decrease of high-frequency movements while sitting during the mindfulness practices in the virtual environment.

**Conclusions:**

With such a small sample size, it is too early to make any specific conclusions, but the presented results may accelerate the use of innovative technologies and challenge new ideas in research and development in the field of mindfulness/telemindfulness.

## Introduction

Attention impairment has often been considered a hallmark of mental illness. Attention training is an important part of meditation, and has proven to augment the ability to sustain attention [1]. Mindfulness as a meditation tool has an important role in psychology, self-awareness, and well-being. The authors Brown and Ryan [2] reported that mindfulness over time was related to a reduction in variable mood and stress in patients with cancer. Mindfulness is an internationally recognized therapy that teaches self-awareness, maintaining own thoughts, sensations, feelings, emotions, and appreciation of your living environment [3]. The mindfulness meditation technique may help patients manage potentially negative outcomes and improve well-being by controlling unselfconsciousness (thoughts on failure). Avoiding problems associated with the future, focusing on the present, being “now,” and controlling the tracking of time may, in addition to well-being, lead to mindfulness. A person who can achieve such an active and open attention state can control thoughts from a distance, free to judge whether they are good or not [4]. In this context, mindfulness can also be considered an important tool for managing anxiety and stress in patients [2]. Kabat-Zinn [3] designed an 8-week meditation course, Mindfulness-Based Stress Reduction, which provides 2 hours of meditation in a group with additional homework. Mindfulness-Based Stress Reduction has demonstrated that awareness of the mind, unconscious thoughts, feelings, and other emotions positively affect major physiological processes and thus decreases the level of stress-related disorders [4-6].

Anxiety and stress disorders can be related to pressure at work, incurable diseases, or neuromuscular disorders, such as Parkinson disease, light traumatic brain injury (TBI), multiple sclerosis, or other diseases of the muscular or central nervous system. Deficits in executive functions, memory, and learning are often documented after TBI. In addition, at least half of those suffering from TBI experience chronic pain and/or sleep disorders, depression, and substance abuse [7].

A review of the literature shows that neural systems are modifiable networks and changes in the neural structure can occur in adults as a result of training [8]. The study reported on anatomical magnetic resonance imaging (MRI) images from 16 healthy meditation-naïve participants who underwent the 8-week mindfulness program [8]. The results obtained before and after the program suggested that participation in a Mindfulness-Based Stress Reduction course was associated with changes in gray matter concentration in the regions of the brain involved in learning and memory processes, emotion regulation, self-referential processing, and perspective taking.

Early rehabilitation in the acute and subacute phase may be a critical period and a key to effective rehabilitation, especially in TBI [9]. A significant drawback is that patients often stay in hospital for a limited time and are soon discharged for recovery at home. Afterward they can visit an outpatients’ clinic. Patients residing close may find the outpatient service convenient, but it could be very inconvenient for those who are in need of ongoing care, are dependent on public transport, or in the worst case do not have access to transport at all. Consequently, external factors such as travel fatigue may hinder the effectiveness of the therapy and, in some, may even increase anxiety and stress. In addition, modern diseases caused by stress and anxiety in the workplace are on the increase, but access to treatment and therapy is usually not possible during working hours [10].

Innovative technologies can ensure real-time communication and data recording/sharing over long distances, even within larger groups of participants [11]. Nowadays, privacy, data security, shyness, and pride are among the most frequent reasons to avoid therapy if a mental disease or neuromuscular disorder is related to work or social status [12].

Some patients prefer to remain anonymous and do not want to reveal their problems, even to colleagues. The sense of “total immersion” created by virtual reality (VR) is an emerging technology that may entirely replace mainstream videoconferencing techniques [13]. These technologies may fulfill patient expectations [14] regarding anonymity and enhance presence [15]. Patients can hide their identify using an avatar and their voices can be disguised. Psychologists and other experts may observe the kinematic changes in motion patterns, gestures, face mimics, and other measurable features [12]. If there is a group, the VR avatars can be synchronized and controlled in real time, using cloud-based technologies. The operator can form groups, deliver individual or group tasks, or lead a private conversation with selected participants. We have developed a technology that is available for home and workplace use, called Realizing Collaborative Virtual Reality for Well-being and Self-Healing (ReCoVR), for which the VR headset is coupled with a mobile phone. The only requirement is a connection to Wi-Fi/4G Internet, plus communication with the cloud server allows remote interaction with other users residing thousands of miles away.

This cloud-based app is used for interaction and communication between a mindfulness expert and participants. Each participant uses a commercially available mobile phone and a simple head-mounted VR headset to join the mindfulness session in the virtual environment (VE). Our main objectives were to design a suitable mindfulness protocol based on Mindfulness-Based Stress Reduction, with tasks in the VE with 360-degree videos, and to test the feasibility of the developed mindfulness/telemindfulness app in a real environment. Additionally, we analyzed head movements during mindfulness sessions to stimulate further initiatives in this research space.

## Methods

### ReCoVR System Design

**Figure 1:**
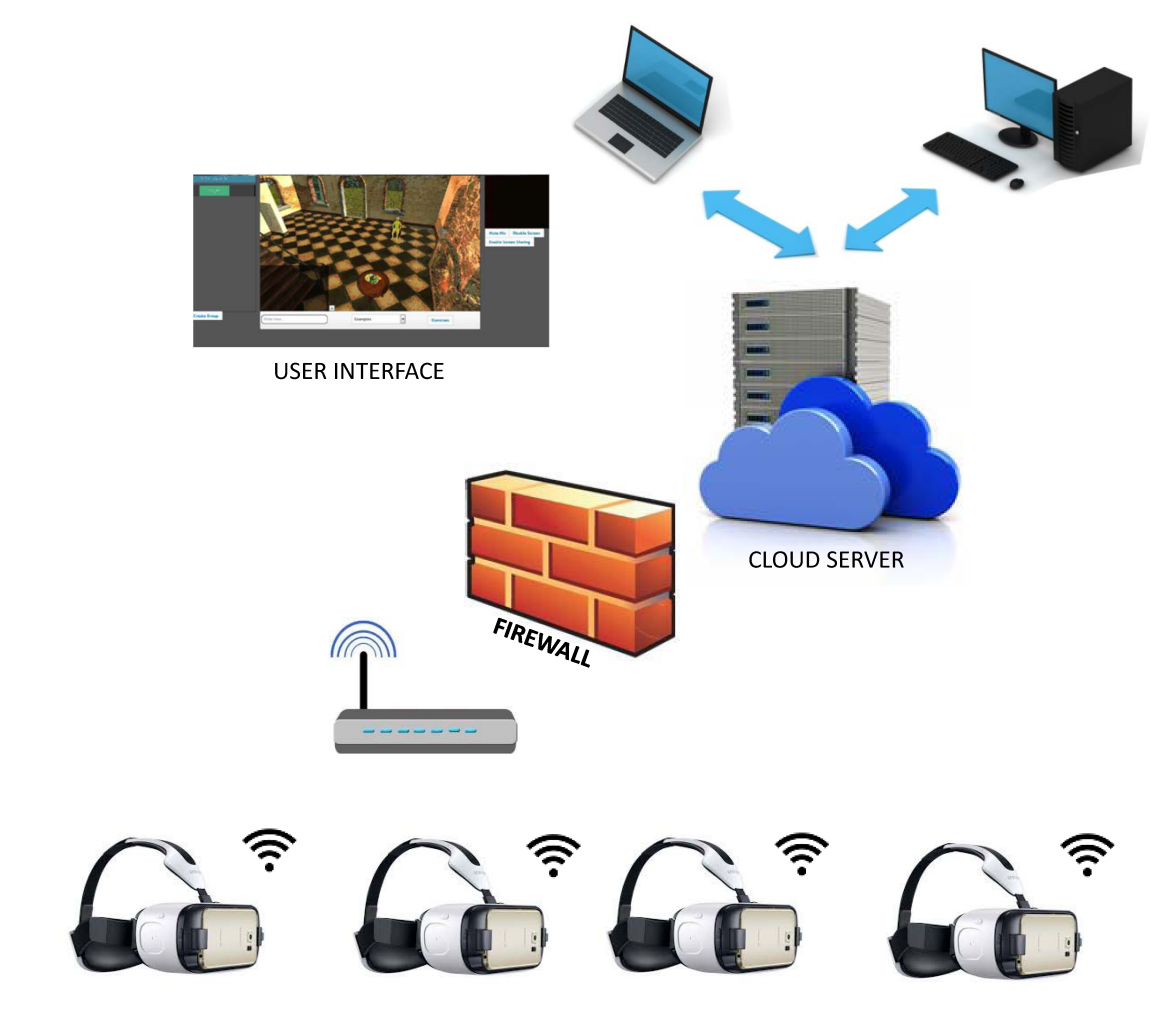
The ReCoVR system consists of a cloud server, serving information for the WebGL scenery and synchronization of the data (audio, video, data) between the server and clients. The clients connect to the server as mindfulness experts (using a computer with Web browser) and as mindfulness therapy participants (using Samsung GearVR 3D headset with Wi-Fi/LTE).

The ReCoVR system for mindfulness/telemindfulness has been designed with two options in mind: self-meditation and remote group sessions (Figure 1). The self-meditation was intended for homework or relaxation at home. The remote group sessions option offered each participant to join the virtual group session and attend a guided mindfulness program lead by the instructor/therapist from a remote location. In both cases, participants used VEs and 360-degree video scenes to carry out the mindfulness program in a seated position. Each participant used a VR headset (head-mounted display), the GearVR (Samsung Electronics Co, Ltd, Seoul, Korea), to interact with the VEs. Various digital objects were designed: VR objects (tables, hourglass), 360-degree videos, music, and natural sounds (birds, wind, waves). The participants set up the options by head movement and pointing on the virtual button. The app for GearVR (tested with Samsung S6, S7, and Note 4 mobile phones) was developed in the Unity3D (Unity Technologies, San Francisco, CA, USA) and built for the Android (tested with Lollipop 5.1 and 6.0 Marshmallow) operating system. The 360-degree videos were recorded at specific locations in County Sligo, Ireland.

Additionally, a server app that hosted the Web-based virtual meeting room (Web user interface [UI]) was developed (Unity3D) and installed on the cloud server (heroku.com). Remote group sessions required a real-time three-dimensional (3D) VR synchronization and also real-time audio-video communication. Our app called a native WebRTC library via Unity3D’s plugin mechanism, in addition to the Android Java NDK / JNI mechanism. Therefore, the Web UI required the availability of the WebRTC in the browser (eg, Chrome or Firefox). The application program interface within the app code could access the microphone and video camera, and relay back real-time audio and video. Both the instructor (Web UI) and the participants (GearVRs) acted as clients and connected to the node.js Web server over Hypertext Transfer Protocol to join the group communication session. Node.js configured the session at the OpenTok server (TokBox Inc). Both clients connected to OpenTok over the WebSocket (Transmission Control Protocol) and used the FIWARE Synchronization Generic Enabler (WebTundra, FIWARE) to synchronize 3D VR data. Both clients pushed their audio (and video for the Web UI) streams and subscribed to the streams of other clients, which were transported over peer-to-peer WebRTC (User Datagram Protocol) connections (Figure 1). The participants provided their GearVR/mobile phone’s unique ID (a security and privacy feature) once before joining the session and connected to the session using any available Wi-Fi/LTE/3G connection.

**Figure 2:**
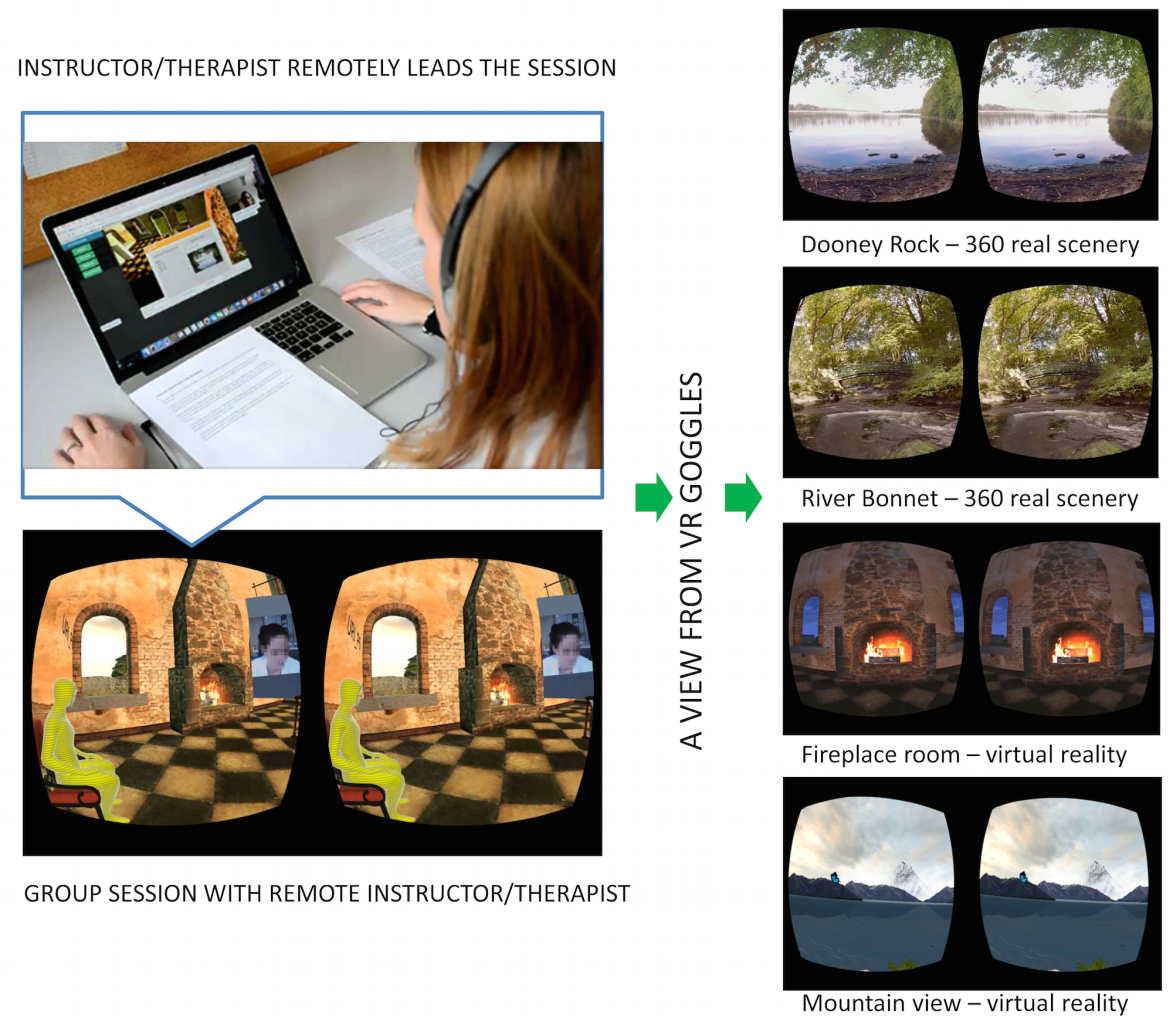
The mindfulness instructor uses the Web interface to manage the group therapy in the virtual room. The Web interface enables video-audio communication with the participants (below left), making subgroups, and assigning tasks (right) for mindfulness sessions. Additionally, the therapist can share documents and lead the session, while everybody can send/receive messages and talk to other group members.

In the self-meditation mode, the user could choose the preferred music, natural sounds, and an environment for mindfulness practice. Four environments were available (Figure 2, right side): two videos recorded in nature with a 360-degree camera (Dooney Rock, a lakeshore in Ireland, and the River Bonnet, a river in Ireland) and two VEs (a dark fireplace room and a bright, wide-open mountain view).

The group session was organized in a virtual room. The user chose to participate in the group session within the 3D VE and received a seat in the virtual fireplace room (Figure 2, lower left). Each participant saw the other participants as avatars and everybody could see the mindfulness instructor on a large screen in the VE. The mindfulness instructor managed the tasks and guided communication over the Web UI (Chrome/Firefox with WebGL support, Figure 2 upper left). The mindfulness instructor saw only the avatars of the participants and a list of their nicknames, whereas the participants saw the mindfulness instructor when his/her video camera was enabled. The mindfulness instructor could also share documents or images, instead of video, or carry out a number of tasks through the Web interface (Figure 2, upper left): (1) video and audio communication with individuals or the entire group, (2) audio communication between the group members, (3) creating additional subgroups of patients, and (4) assign or interrupt exercises to each individual or group. The choice of the exercises and tasks (Dooney Rock, River Bonnet, fireplace room, or mountain view) was determined by the mindfulness program.

### Mindfulness Virtual Reality Program

**Figure 3:**
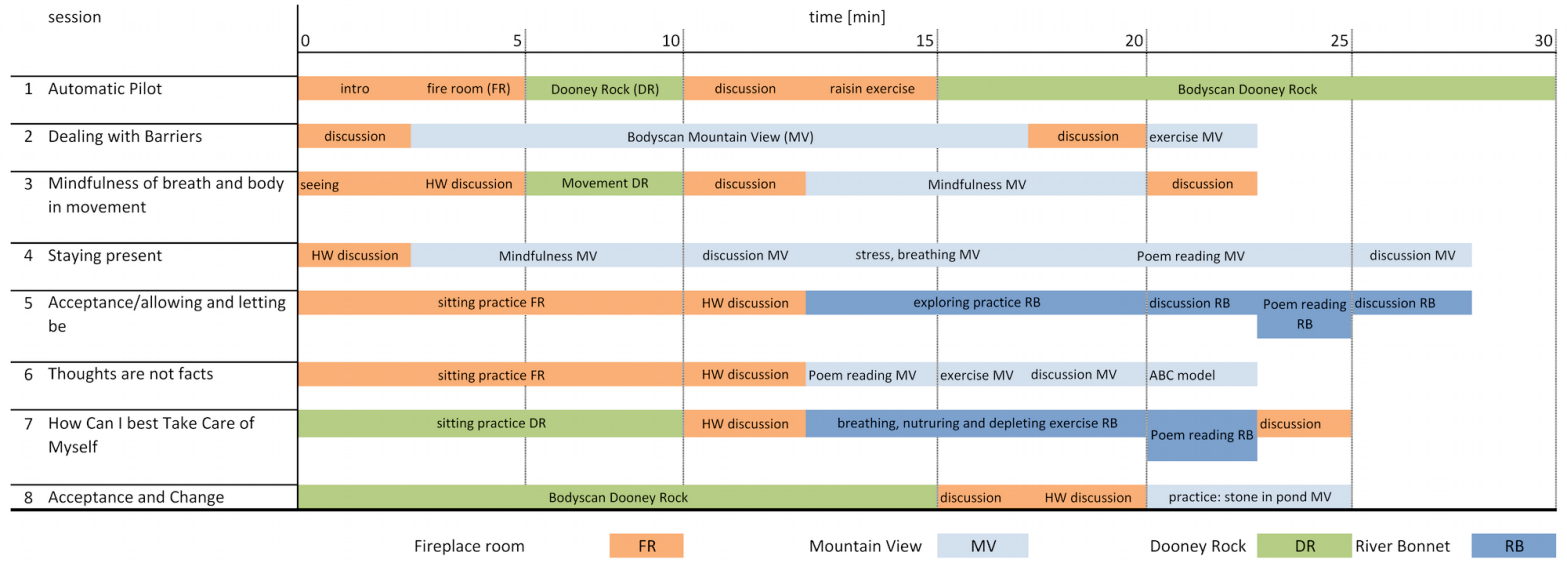
Modified GearVR group mindfulness program.

The 3D VR head-mounted devices were not suited for several hours of use, as would be required by the designed Mindfulness-Based Stress Reduction [3,6]. Participants could suffer from headaches or other tolerance issues from overuse of the VR headset. Additionally, it was noted there was a technical limitation in displaying comprehensive VR graphics—overheating of the mobile phone [16]. The overheating of the mobile phone caused unacceptable degradation of graphics and communication quality. Therefore, we limited the sessions to 30 minutes, a time acceptable from the tolerance point of view and the overheating was not excessive. Due to the mobile phone overheating issues [16], tolerance to VR and technology compliance, we designed a novel mindfulness VR program and split the mindfulness program into sessions (Figure 3). Activities within each session were compliant with the designed VE.

#### Session 1: Automatic Pilot

The first session started in the virtual fireplace room with all participants seated as avatars and listening to the mindfulness instructor. In the first five minutes, the instructor presented basic rules about respect, confidentiality, and how to take care of themselves. Afterward, the instructor changed the VE for all participants to Dooney Rock and carried out an exercise called “Stone in the Water,” then reverted back to the virtual fireplace room and led the discussion for 3 minutes before continuing with another exercise called “Tasting the Raisin.” Session 1 finished with a 15-minute body scan exercise, which was a relaxing meditation. The participants listened and followed the text read by the instructor.

#### Session 2: Dealing With Barriers

The second session also started in the virtual fireplace room with 3 minutes of discussion followed by the same body scan as in session 1, but this time in the mountain view VE. Afterward, the participants returned to the virtual fireplace room and had a discussion for 3 minutes, before finishing the session back in the mountain view VE with a “thoughts, feelings, and sensations” exercise.

#### Session 3: Mindfulness of Breath and Body in Movement

The third session also started in the virtual fireplace room with a 3-minute “seeing and hearing awareness” exercise and discussion, followed by a “movement practice” in the Dooney Rock VE. Discussion was led again in the fireplace room for 3 minutes before a “breath and body mindfulness” exercise was carried out in the mountain view VE. The session finished with further discussion in the fireplace room.

#### Session 4: Staying Present

The fourth session started with 2 minutes of discussion in the fireplace room followed by the following exercises in the mountain view VE: “mindfulness breath, body, sound,” “stress reaction/response,” “breathing space,” and poem reading (*Wild Geese* by Mary Oliver). The session finished with a discussion, this time in the mountain view VE.

#### Session 5: Acceptance/Allowing and Letting Be

The fifth session started in the fireplace room with a 10-minute sitting exercise, and was followed by a discussion, “exploring difficulty practice,” and a poem reading (*Wild Geese* by Mary Oliver), all in the 360-degree video of River Bonnet. The session finished with a discussion, this time in the River Bonnet VE.

#### Session 6: Thoughts Are not Facts

The sixth session started in the same way as session 5 in the fireplace room and continued in the mountain view VE with a poem reading, a “moods, thoughts, and alternative viewpoints” exercise, and finished with an exercise called the “ABC Model.”

#### Session 7: How Can I Best Take Care of Myself?

The seventh session started with a sitting exercise for 10 minutes, but this time in the Dooney Rock VE. Afterward, the group had a discussion in the fireplace room. The mindfulness therapy continued with a “breathing space” exercise for 3 minutes, “nurturing and depleting” exercise for 5 minutes, and a poem reading (*Summer’s Day* by Mary Oliver) for 2 minutes, all in the River Bonnet VE.

#### Session 8: Acceptance and Change

The final session started with a 15-minute long body scan in the Dooney Rock VE, followed by discussion in the fireplace room for 5 minutes, and finished with “Stone in the Pond,” a reflection on what was learned exercise, in the mountain view VE [17].

### Psychometric Tests

The modified mindfulness VR program’s effect on participants’ health and well-being was measured by two psychometric tests: the Satisfaction With Life Scale (SWLS) and Mindful Attention Awareness Scale (MAAS). The SWLS contains five items and factor loadings [18], each scored using a scale from 1 to 7 points. The participants were asked to honestly indicate their agreement with each item by putting the appropriate number in front of the statement: 1=strongly disagree, 2=disagree, 3=slightly disagree, 4=neither agree nor disagree, 5=slightly agree, 6=agree, and 7=strongly agree. The sum of items presented the SWLS score: 31-35=extremely satisfied, 26-30=satisfied, 21-25=slightly satisfied, 20=neutral, 15-19=slightly dissatisfied, 10-14=dissatisfied, and 5-9=extremely dissatisfied.

The MAAS is a 15-item scale [2] for assessing the characteristics of a disposition toward mindfulness (ie, open or receptive awareness of and attention to what is taking place in the present). The MAAS was validated with college, community, and cancer patient samples [19] and is considered a strong psychometric indicator. The scale contains statements about everyday experience that should be rated honestly on a 6-point scale: 1=almost always, 2=very frequently, 3=somewhat frequently, 4=somewhat infrequently, 5=very infrequently, and 6=almost never. The answers should reflect one’s experience rather than what the participant thinks his experience should be. The mean of the 15 items presents the MAAS score; a higher score means a higher level of dispositional mindfulness. The skilled mindfulness instructors can finish the test in less than 10 minutes.

Comparisons (mean, standard deviation) of data assessed with the psychometric tests before the mindfulness sessions, midterm, and after the mindfulness program were carried out separately for employees and patients. Additionally, each participant’s results were examined to confirm or drop the deviation from the group mean.

The participants with cognitive disorders also took the Mini-Mental State Examination (MMSE), which is a questionnaire that consists of 30-point questions and is mostly used in clinical settings to measure cognitive impairment. Often it is used by clinicians to screen for dementia [20]. Scores between 25 and 30 are considered normal, 24 and 21 as mild, 20 and 10 as moderate, and less than 10 as severe impairment, according to the National Institute for Health and Care Excellence.

### Head Motion Analysis

Head motion was monitored throughout the sessions using the mobile phone’s built-in orientation sensor. To avoid singularities due to “gimbal lock,” data were supplied in quaternions [21] with a timestamp:

q=[q_0_ q_1_ q_2_ q_3_]^T^

q=q_1_^i^+q_2_^j^+q_3_^k^+q_4_

**Figure 4:**
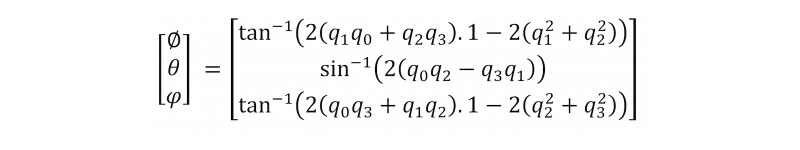
Equation for transformation to the Euler angles.

**Figure 5:**
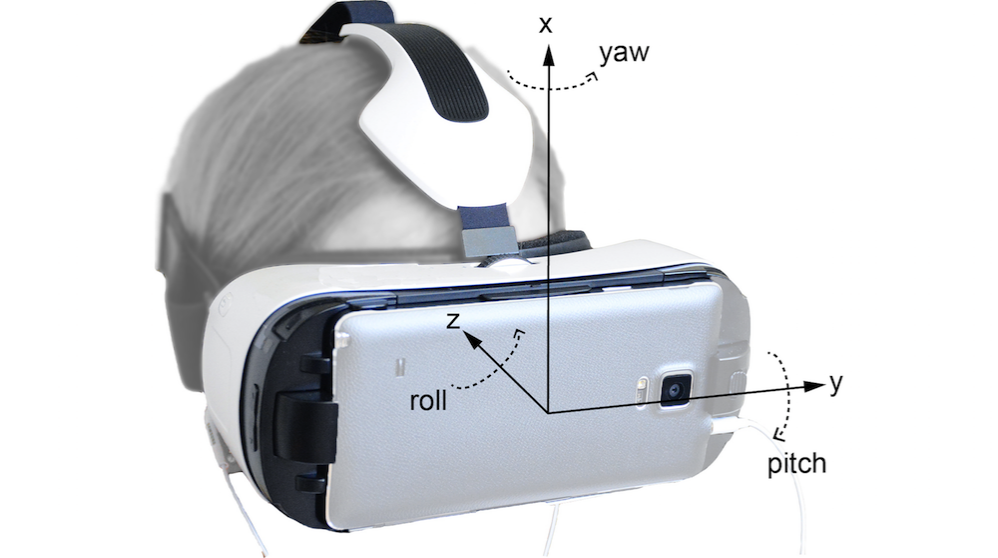
Coordinate frame (YZX) attached to the GearVR was defined by Samsung mobile phone and its built-in sensors. The Euler angles yaw, pitch, and roll were used to present head motion.

The transformation to the Euler angles was carried out similarly to the rotation operation on the selected vector [21], presented as Euler angles ε=[Φ,θ,φ]^T^ in the rotation sequence YZX (Figures 4 and 5) (yaw, pitch, roll).

To avoid limitation to ±π/2 with arctan function atan2 function, the SpinCalc package for Matlab (Mathworks Inc, Natick, MA, USA) was used. The obtained signals were preliminary filtered (Butterworth, f=0.5 F_S_, F_S_=50 Hz) and discontinuities were detected with wavelets [22] and eliminated (using findpeak function of Matlab and own functions for elimination).

During the mindfulness sessions, head motion was constantly monitored. The head motion (roll, pitch, yaw) within the session as well as across the sessions with similar activities (body scan, mindfulness, or poem reading in the same VE) was analyzed using Matlab. The signal analyses were carried out in both the time and frequency domain; the amplitude of the head motion in 3D space and the frequency analysis of the head rotation, yaw. Fast Fourier transform (FFT) was used for spectral analysis of the head motion (yaw) signal (Signal processing Toolbox, Mathworks Inc). In particular, the high frequencies above the cut-off frequency were carefully examined. The FFT specters were compared between the sessions with similar content. The assessment of the section body scan in Dooney Rock from session 1 was compared with the data of the section with the same name from the final session. Further, data obtained from the sitting practice exercises in the fireplace room at the beginning of sessions 5 and 6, and data obtained within session 7, were compared for employees and patients, respectively.

The Euler angles (yaw, pitch, and roll) for similar sections of particular sessions, as previously described, were presented in 3D graphs. Additionally, the motion during the poem reading tasks and River Bonnet virtual worlds were compared between the fifth and sixth sessions. The outcomes were quantitatively evaluated by calculating the entire motion range (the estimated area of the yaw-roll plane curve) of the head for the compared sessions.

### Participants

The feasibility study involved eight participants in total divided into two groups: four persons of different professions were volunteers/employees of the hospital, aged between 27 and 40 years, with self-reported anxiety and stress. The other four participants were outpatients aged between 24 and 48 years; one patient had a brain tumor and the other three were TBI patients. The medical professionals reported that patients suffered from anxiety and stress due to their uncertainty of the neurorehabilitation program’s outcome. These participants were recruited during their visit to the outpatient hospital only if they had matched the inclusion criteria. The inclusion criteria for patients consisted of mild or no cognitive impairment to be able to understand the instructions and follow the program. High diopters were exclusion criteria because the equipment was limited to approximately –3.5 diopters. The headset was not suitable for those with astigmatism and who wore glasses.

The study was approved by the University Rehabilitation Institute’s Ethics Committee and all participants signed the written consent that is compliant with the Declaration of Helsinki and European Convention on Human Rights.

### Protocol

The feasibility study observed the proposed mindfulness VR program for eight consecutive weeks. The psychometric tests were carried out by the mindfulness instructors before and after the mindfulness sessions (Table 1). Additionally, midterm scoring was performed at week 4. The participants with cognitive disorders were screened for MMSE by a clinical professional at the outpatient hospital. Every week, the participants attended one VR-supported mindfulness group session (Table 1) for approximately 25 minutes, with a 2-minute guided breathing exercise. Additionally, the participants were given instructions for homework: from upright and alert posture, simple breathing and relaxation exercises, and conversations with a friend.

Group sessions were carried out in the hospital environment and under the supervision of the physician/psychologist. Each participant sat in a comfortable chair in a separate room and used the developed software, running on a GearVR, to interact with the group. The equipment was connected to a Wi-Fi or LTE wireless network if the Wi-Fi signal was weak or unavailable to connect to the cloud server (Figure 1). The mindfulness instructor led the sessions remotely from her office. However, before the mindfulness program started all participants were instructed how to properly use the equipment and how to react in case of malfunctioning, etc.

At the end of the mindfulness VR program the participants filled out a short questionnaire; the questionnaire for patients and employees contained questions on the GearVR interface, and the questionnaire for the mindfulness instructor contained questions that related to the Web use interface. Both groups answered voluntarily and evaluated the statements in the questionnaire on a Likert-type scale (1=disagree, 2=light disagree, 3=equal, 4=agree, 5=strongly agree).

**Table 1:** The mindfulness study protocol.

Week	Tests
1	MAAS [2]; SWLS [18]; MMSE (for patient only)
1-4	25 minutes mindfulness VR per session; 2 minutes breathing exercise per session; 1 session/week for 4 consecutive weeks; homework
4	Psychometric tests
4-8	25 minutes mindfulness VR per session; 2 minutes breathing exercise per session; 1 session/week for 4 consecutive weeks; homework
8	Psychometric tests

**Figure 6:**
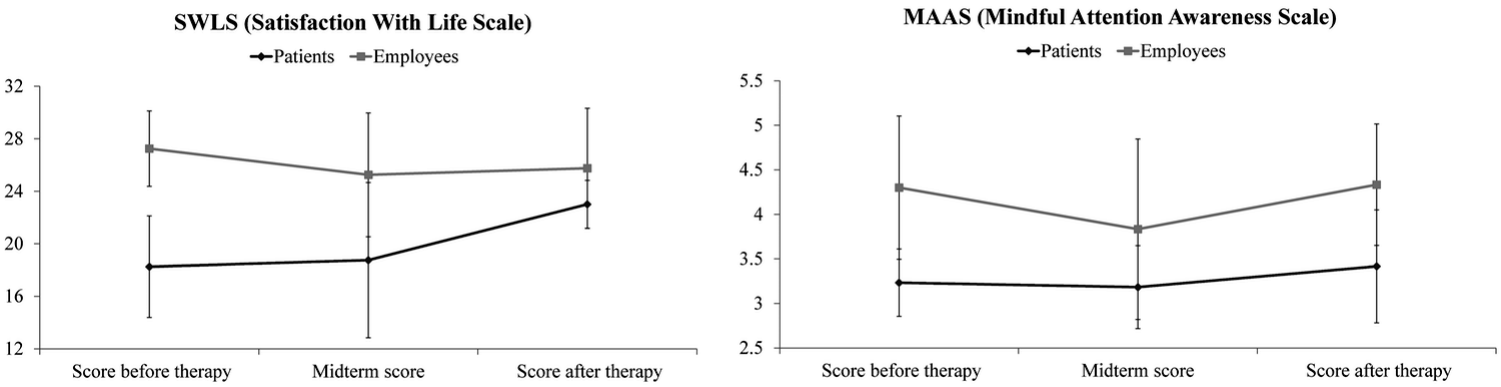
The psychometric tests results.

## Results

The scores of the SWLS (Figure 6, left) showed that most of the participating patients varied between slightly satisfied and dissatisfied levels of “satisfaction with life” (mean 18.3, SD 3.9) at the beginning of the mindfulness VR program and achieved higher levels of satisfaction with life at the end of the program (mean 23.0, SD 1.8). The participating patients achieved more points on the MAAS (Figure 4, right) at the end of mindfulness VR program (mean 3.4, SD 0.6 vs mean 3.2, SD 0.4). However, a reduction in MAAS points was noticed at the midterm assessment (mean 3.2, SD 0.5). The results in Table 2 reveal that only patient 1’s score (MAAS=2.3) deviated the most from the mean value. More detailed insight revealed that items 8 (“I rush through activities without being really attentive to them”) and 15 (“I snack without being aware that I’m eating”) were unexpectedly lower. However, the patient’s SWLS score increased from 16 (slightly dissatisfied) to 21 (slightly satisfied). Only one patient (patient 2) had an MMSE score less than 30; it was 19 when entering the program, but the cognitive score was 26 at midterm and 29 when the program finished. In general, the employees were satisfied with their life (SWLS mean 27.3, SD 2.8), despite their SWLS decreasing slightly to mean 25 (satisfied). However, this deviation was because one employee scored 21 (slightly satisfied). This employee also had a lower MAAS. Generally, the employees kept their aggregate MAAS score at the same level (mean 4.3, SD 0.7) as at the beginning of the mindfulness VR program (mean 4.3, SD 0.8). At the midterm, their MAAS score dropped (mean 3.8, SD 1.0).

**Figure 7:**
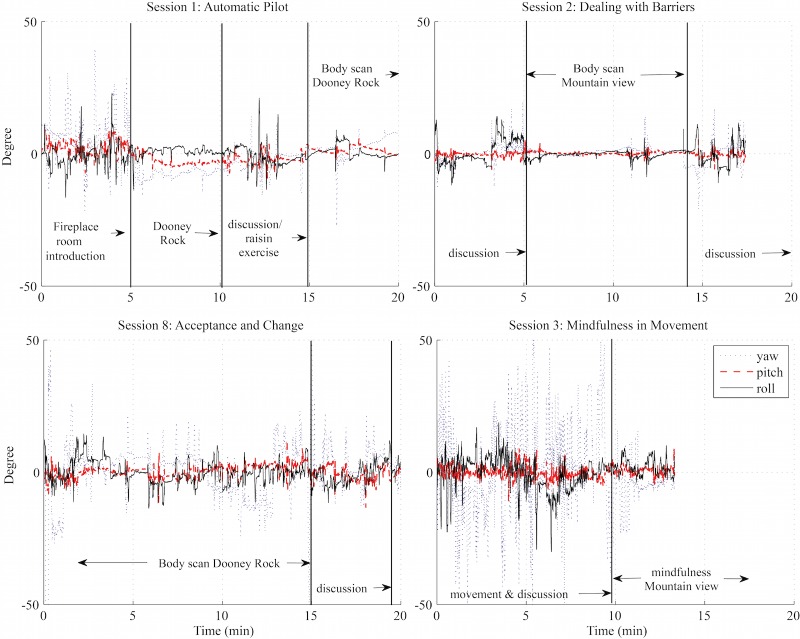
Head motion (pitch: head up and down; roll: head tilt left and right; yaw: head rotation left and right) during selected mindfulness program sessions.

Head movements (Figure 7) were analyzed separately for each session and task (VR or 360-degree video) in the 3D space. Practically, we noticed only small amplitudes (less than ±10 degrees) of up and down head movements (pitch) more or less in relation to the frequent glimpses between the instructor view and the surrounding area. Consequently, the rotations of the head (yaw) were of a larger scale (up to ±40 degrees). Roll/tilt of the head was of a larger scale only when movement exercises were requested from the participants (Figure 7, lower right), but most of the time such movements were within a small range (less than ±10 degrees). Also, the rotations of the head were small during the mindfulness sessions in VR (Figure 5, upper right mountain view) and 360-degree videos (Figure 7, Dooney Rock).

**Figure 8:**
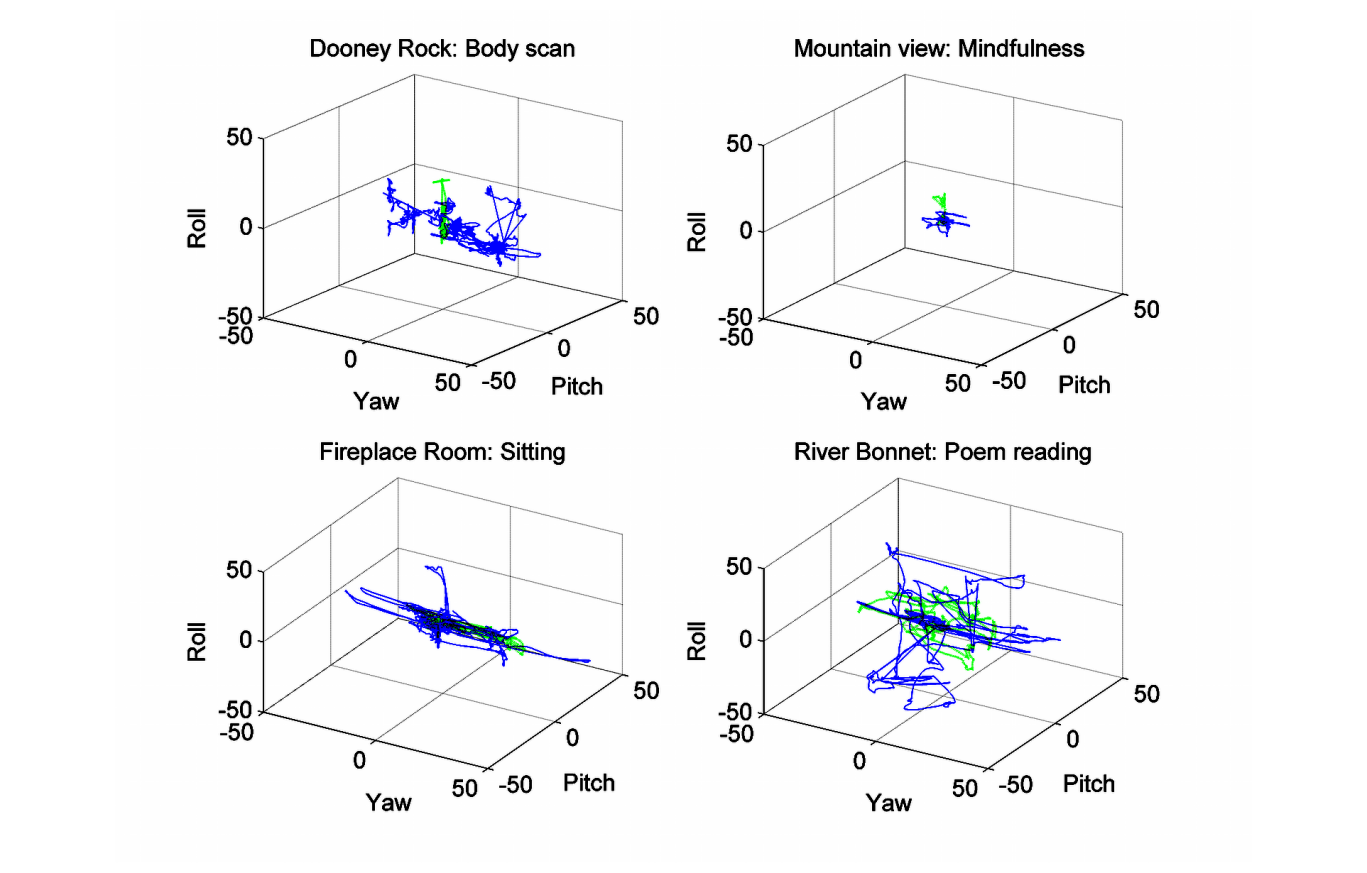
The 3D head movements during various VR and 360-degree video tasks for employees. The solid blue line represents the previous session and the green dotted line the following session, according to the mindfulness program.

**Figure 9:**
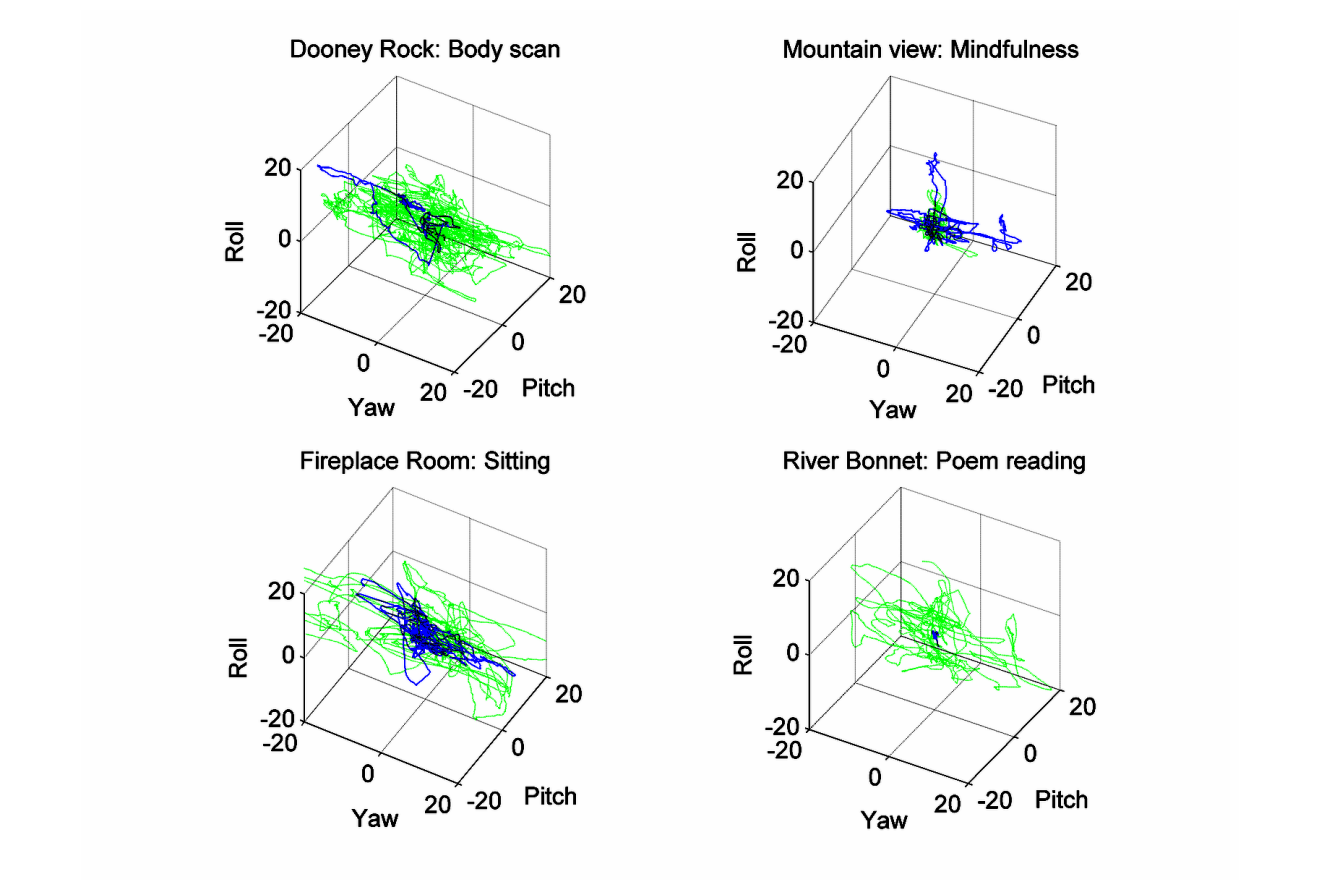
The 3D head movements during various VR and 360-degree video tasks for patients. The solid blue line represents the previous session and the green dotted line the following session, according to the mindfulness program.

In general, the employees rotated their heads left and right to observe the shore of the lake (Figure 8, Dooney Rock, blue solid line). This scenery was obviously very attractive only in session 1, whereas at session 8, there was hardly any head rotation (Figure 8, Dooney Rock, green dotted line). The opposite behavior was shown by the patients. Not only did they rotate their heads, but they also moved up and down to see the trees and the surroundings of the lake during the body scan session in the Dooney Rock 360-degree scenery. However, the range of motion was at least half the mean range shown from the employees (Figure 9, Dooney Rock). The mindfulness practice in the VR mountain view scenery (Figure 8) was rather calm, with slight head rotation (less than ±10 degrees), which was weaker in session 3 than in session 2. The patients (Figure 9, mountain view) performed almost the same action with one exception. In session 2, they also tilted (roll) their head mostly to the left. During sitting in the virtual fireplace room (Figures 8 and 9, fireplace room), both groups—the employees and the patients—rotated their heads left and right to see the virtual fireplace, avatars of the other participants, and from time-to-time the instructor/therapist’s video session. The employees reduced their motion curve area by 23% in session 6 compared to session 5, whereas the patients increased their motion curve area by 17% in session 6. However, the patients achieved about half the range of motion of the employees (Figure 8, fireplace room). The head movements during the poem reading in session 5 at the virtual River Bonnet revealed that almost all patients calmed down and some of them also fell asleep for up to 2 minutes (Figure 9, River Bonnet, blue line). In session 7, they demonstrated head rotation (up to ± 20 degrees) and head tilt (up to ± 10 degrees). It was an opposite result with the employees, who demonstrated head movements in all directions (up to ± 15 degrees) in session 5, but a week later were calmer (motion curve area smaller for 72% with motion up to ± 10 degrees head rotation).

**Figure 10:**
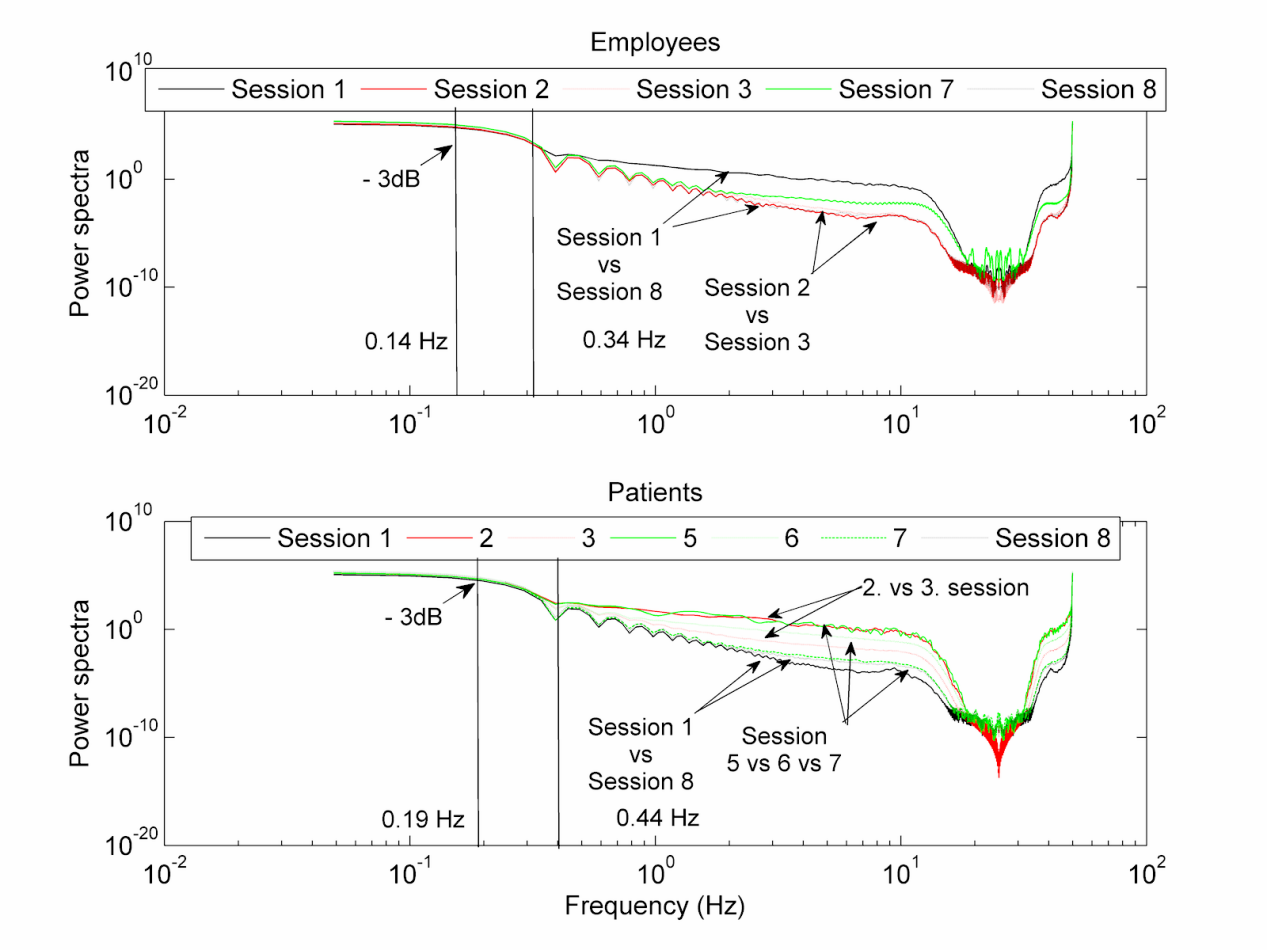
Power spectrum (FFT) of head movements during specific tasks (VR and 360-degree video) of the comparable mindfulness sessions. The patients demonstrated more calm head movements during VR tasks, but the employees did in the 360-degree video tasks.

The head rotation during the body scan in the virtual Dooney Rock in session 1 was compared with the recordings of the same task in session 8, in the frequency domain. The employees demonstrated considerable reduction of high-frequency movements greater than 0.34 Hz. However, the observed differences greater than 0.44 Hz assessed in patients were negligible (Figure 10).

Conversely, a comparison of sessions 2 and 3 in the mountain view VE did not show any differences for employees, yet considerable reduction of high frequencies greater than 0.44 Hz for patients (Figure 10, bottom). Additionally, a comparison of the power spectra of the head movements during the sitting mindfulness practices in sessions 5, 6, and 7 also revealed a gradual decrease of high-frequency movements. These turbulent head movements greater than 0.44 Hz had very low amplitudes in session 7. There were practically no changes noticed in sessions with employees. All three sitting practices in the fireplace room VE had the same power spectrum as session 7.

**Figure 11:**
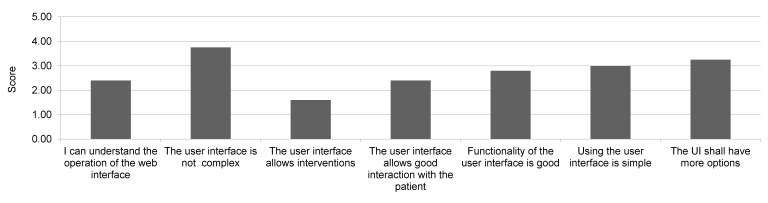
Evaluation of user interface by employees/mindfulness instructor (Likert-style scale, 1=disagree, 2=light disagree, 3=equal, 4=agree, 5=strongly agree).

**Figure 12:**
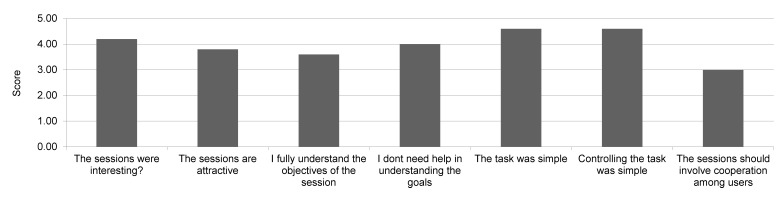
Session/task ratings by patients (Likert-style scale, 1=disagree, 2=light disagree, 3=equal, 4=agree, 5=strongly agree).

The mindfulness instructor/employees agreed that the UI was not complex, and was easy to use, but did not yet allow enough interventions and should have more options (control buttons) available (Figure 11). The patients also agreed that the game was simple (score 4.6) and that UI was not complex (score 4.4), but they were not convinced whether the users should cooperate and interact with one another within the tasks (Figure 12).

**Table 2:** The outcomes of the study for each individual participant.

Participant			Before	Midterm	After
**Patient**					
	**1**				
		MAAS	3.3	2.3	2.3
		SWLS	16	18	21
	**2**				
		MAAS	4.2	4.1	4.8
		SWLS	16	11	24
	**3**				
		MAAS	3.1	3.4	3.6
		SWLS	17	21	22
	**4**				
		MAAS	2.4	2.5	3.0
		SWLS	24	25	25
**Employee**					
	**1**				
		MAAS	3.3	2.5	3.9
		SWLS	28	22	25
	**2**				
		MAAS	4.2	3.5	3.7
		SWLS	25	22	21
	**3**				
		MAAS	4.5	4.5	4.5
		SWLS	25	25	25
	**4**				
		MAAS	5.2	4.7	5.2
		SWLS	31	32	32

## Discussion

Patients found the novel technology rather easy to use and not too complex. They were also satisfied with the mindfulness VR program. Much more skeptical were the group of employees, who may not have had such high expectations as the participating patients. They felt that the Web UI should allow more control options, but both groups agreed that the system was not complex and was easy to use. Occasionally, individuals had some difficulties in understanding the goals they were required to achieve. Furthermore, the study revealed how critical the major weakness could be of the proposed system: the mobile phone overheating, a known issue of the Samsung mobile phones [16]. The annoying consequences of the overheating were a gradual loss of graphic detail and disrupted voice communication. In spite of the mobile phones used in the GearVR being the ones with the highest display resolution currently available on the market, the participants were not able to read the text of the documents provided in the virtual room. However, they could follow the photos and video and the text was then read by the instructor.

The employees participating in the study had high SWLS scores. They were all slightly satisfied with their life except one participant, who was extremely satisfied with their life, according to the applied scale. The score has dropped for 2 grades. The MAAS score dropped in the midterm, due to two participants seeming to be preoccupied with their future or job. One of them resolved this problem with the therapy and the overall group score returned slightly above the initial level. The participating patients suffering from neuromuscular diseases or recovering after TBI were all slightly dissatisfied with their life [23]. Yet the therapy resulted in a much higher SWLS end score. Furthermore, the low standard deviation at the end of the therapy pointed to a moderate satisfaction with life from all participants. Additionally, all patients took benefit from the therapy in terms of higher MAAS score, except one participant. A detailed analysis of the scale items revealed that this patient had improved memory function, but still had difficulties focusing on the present. This patient spent most of their time at home and was having family matters. This patient was also the only one with a lower MAAS score at the end of the mindfulness program, although the patient still had a higher SWLS score advancing from “slightly dissatisfied” to a “slightly satisfied” level. In particular, we also noticed that two patients had a slight decrease in MAAS at midterm, which was not surprising. Patients were often confused during the therapy and unsure of the success of the therapy [24]. These promising results at the end of the proposed mindfulness program may indicate reduced subjective pain and changes in the brain [25-27]. However, the authors were also aware of the limitations: several interactions between meditation training and order effect. Yet we had to consider that “no treatment” was not an acceptable option and could be considered as unethical. However, long-term mindfulness practice may lead to emotional stability that may help get over psychological disorders [28]. It was also reported that practicing mindfulness with possible negative emotional events invoked actuation of specific brain regions associated with emotions [29]. The authors reported that the group, in attending mindfulness sessions, showed increased activation in prefrontal regions as identified by functional MRI and, therefore, this group attenuated their emotional arousals more easily compared to the control group [1]. Similar findings on functional reorganization of the brain for focused attention by meditation-related practice were reported by Manna et al [26]. However, we cannot generalize that all types of meditation or mindfulness practice would result in similar clinical effects [30]. For example, the most popular mindfulness program, Mindfulness-Based Stress Reduction, results in positive effects on psychological well-being and causes changes in the gray matter density in certain regions of the brain. Consequently, the learning process, emotion regulation, and self-respect may improve [8].

Mindfulness training involves various interventions for helping people to overcome their fears and neuropsychological disorders. Some of the often-applied mindfulness instructions also invoke contemplative or mindful movements [31]. For example, awareness of sensation used sensations related to autonomic response, even kinematics, and awareness of the present moment (also applied in our mindfulness program) incorporated body movement sensation and breathing but staying in the present moment, not lost in the past or future. Therefore, we directly measured the changes in head motion as a consequence of being present in the VE or 360-degree video providing mindfulness instructions. There have been several theories, as mentioned by Russell and Arcuri [31], trying to explain the potent attentional focus as a consequence of movement and the benefits of mindfulness training on working memory. We are not able to provide enough insight into these mechanisms. Yet our results suggest that patients with neurophysiological and/or neuropsychological disorders increased their head movement amplitudes when they took mindfulness training in the same environment for the second time, although the amplitudes of the movements were in the range of ±20 degrees, approximately half the movement range that employees had. This may be compliant with the suggestions [31,32] that a combination of movements with mindfulness meditation training may engage working memory and be effective in attention focusing. On the contrary, the participating employees in our study reduced their movement amplitudes when they took mindfulness training in the same environment for the second time. The explanation for these differences could be that patients were more instruction dependent, especially awareness of sensation and awareness of the present moment [31], than employees, who after their first relaxation sessions became calm and less interested in the VE. This statement was supported by the movement analysis in the frequency domain. The employees reduced their higher frequency (>0.3 Hz) movement between the first and last session of the body scan exercise in the Dooney Rock environment, whereas there were almost no differences in the movements in the mountain view VE. By contrast, with the patient group we could not find any difference in movements greater than 0.4 Hz when doing the body scan exercise in the Dooney Rock VE, but considerably more differences appeared when sitting in the fireplace room VE. These findings may suggest that the VE can be more effective for patients and 360-degree video for intact persons with mild psychotic problems. However, because awareness is hard to measure, this could be further confirmed using the clinical and cognitive outcomes [31].

Hereinafter the proposed measurements of head motion could be used as a tool for the assessment of the outcomes of different strategies and mindfulness training instructions. In this way, the mindfulness instructor could alter the instruction set for the next mindfulness sessions. Perhaps a rough estimation of stress reduction, anxiety, or SWLS would have been possible if a correlation with a large group of patients with neuropsychological disorders was demonstrated.

The proposed technical solution for telemindfulness VR has proven to be easy to use and user friendly, and may become useful for remote mindfulness training for various groups of users: hospital patients with neuropsychotic disorders, employees of large enterprises who are not able to leave their offices, and people residing far from the centers where mindfulness is practiced within groups. We managed to demonstrate the feasibility of the proposed solution in a limited group of participants, yet we are aware that some mindfulness training outcomes are extremely difficult to measure [33]. Therefore, the SWLS and MAAS tests were applied and revealed that all but one made a good use of the mindfulness VR training. Additionally, the developed prototype can provide measurable data that may be used in future to predict participant behavior [12] or reaction to specific mindfulness instructions. This leads to further challenges and generation of new ideas in terms of the technology use and research that can continue the development of mindfulness/telemindfulness and its effects from a clinical perspective.

## Abbreviations

FFT: fast Fourier transform
MAAS: Mindfulness Attention Awareness Scale
MMSE: Mini-Mental State Examination
ReCoVR: Realizing Collaborative Virtual Reality for Well-being and Self-Healing
SWLS: Satisfaction With Life Scale
TBI: traumatic brain injury
UI: user interface
VE: virtual environment
VR: virtual reality
